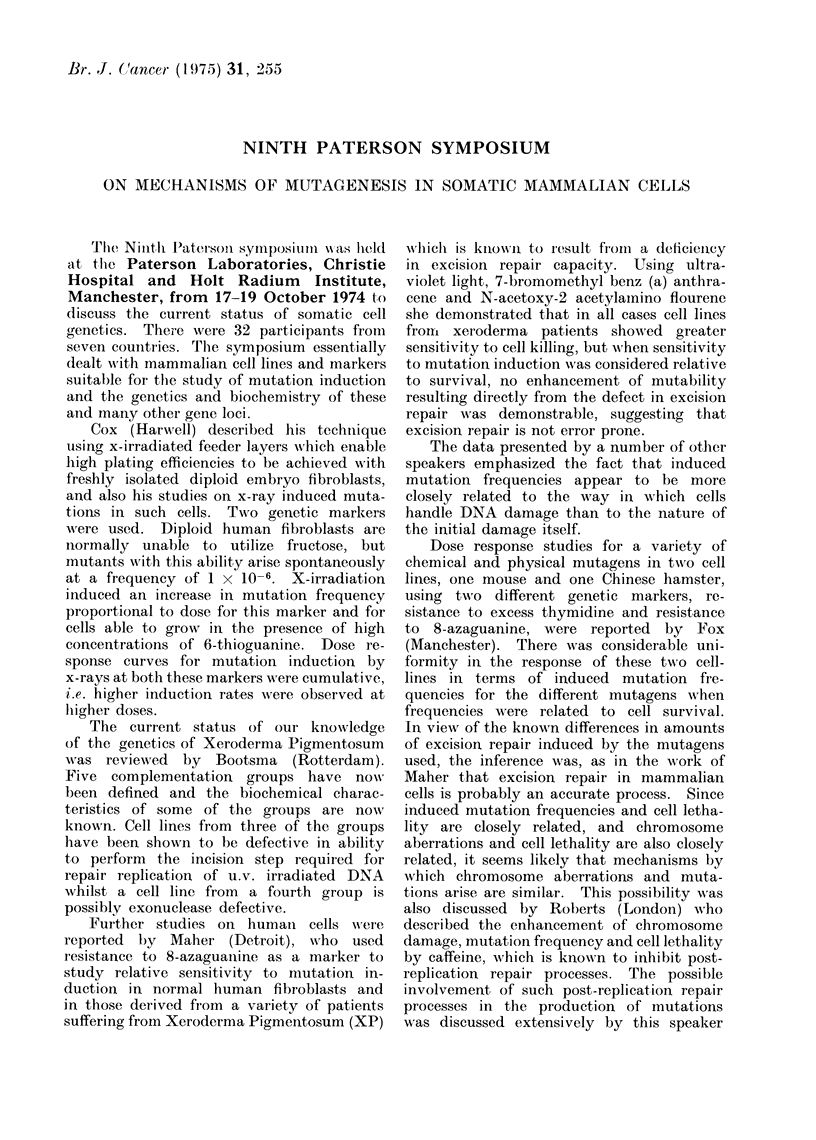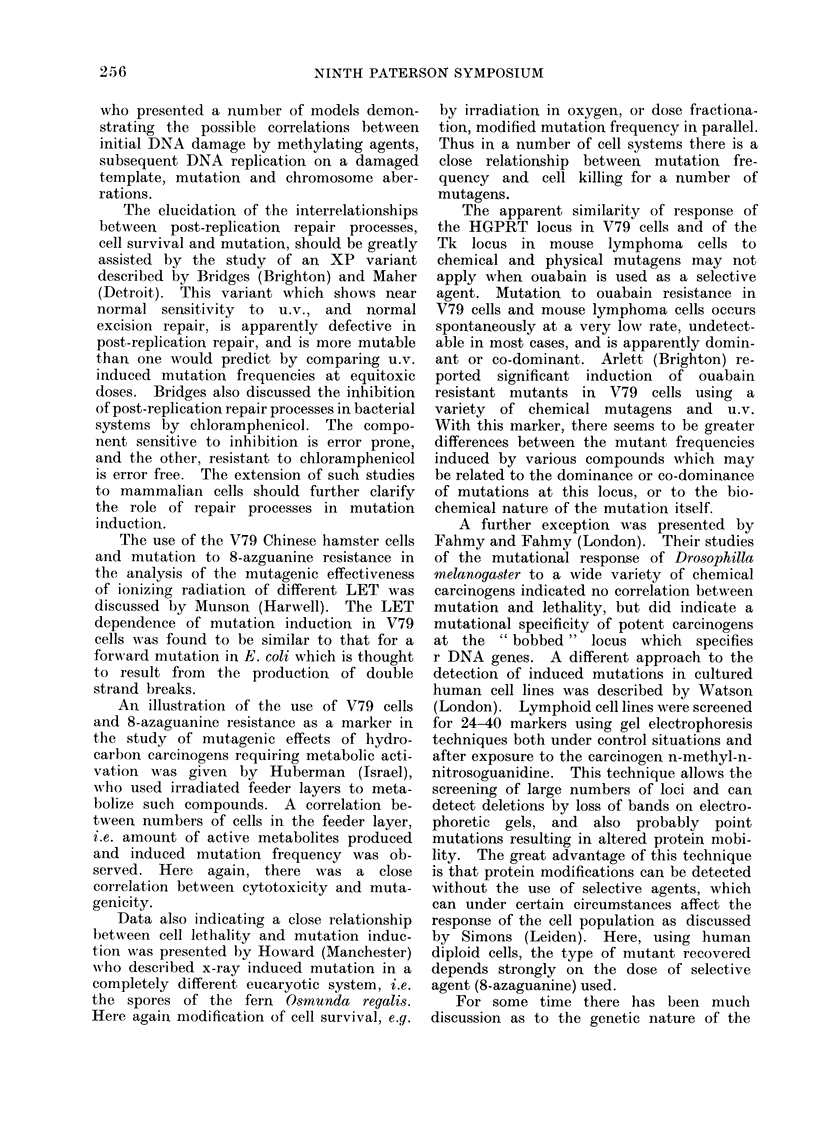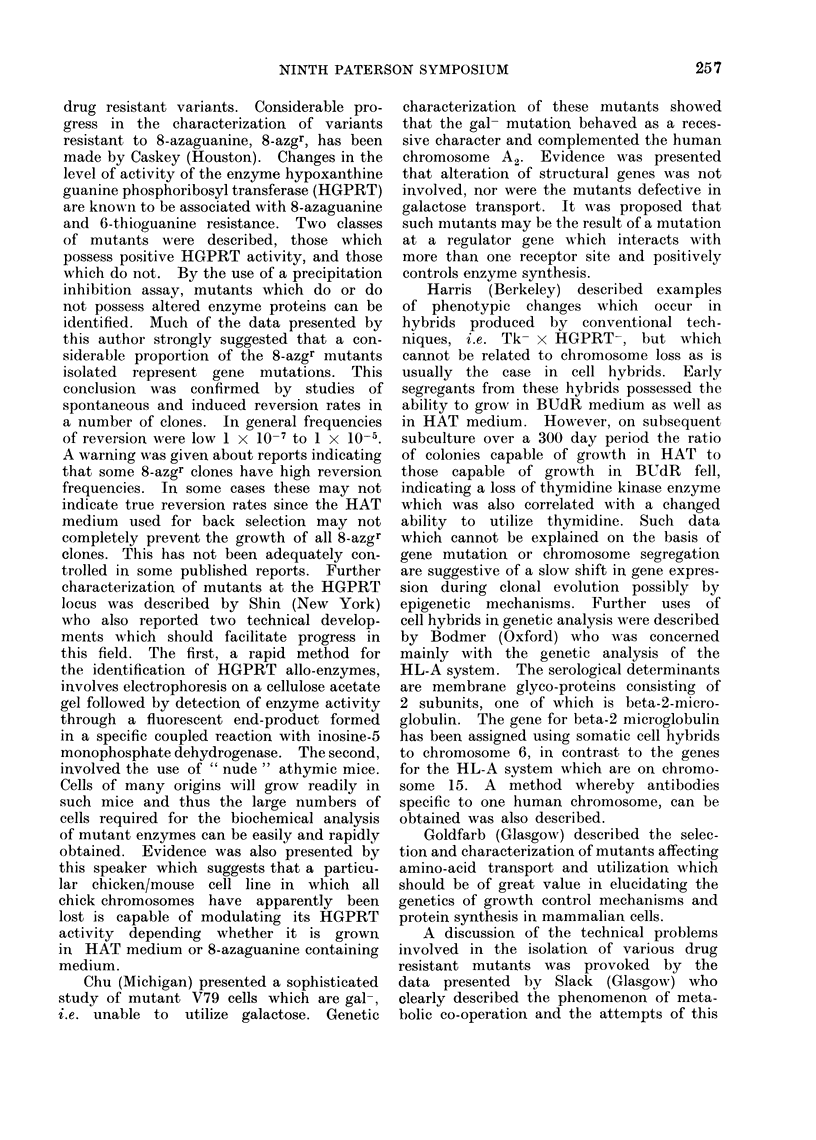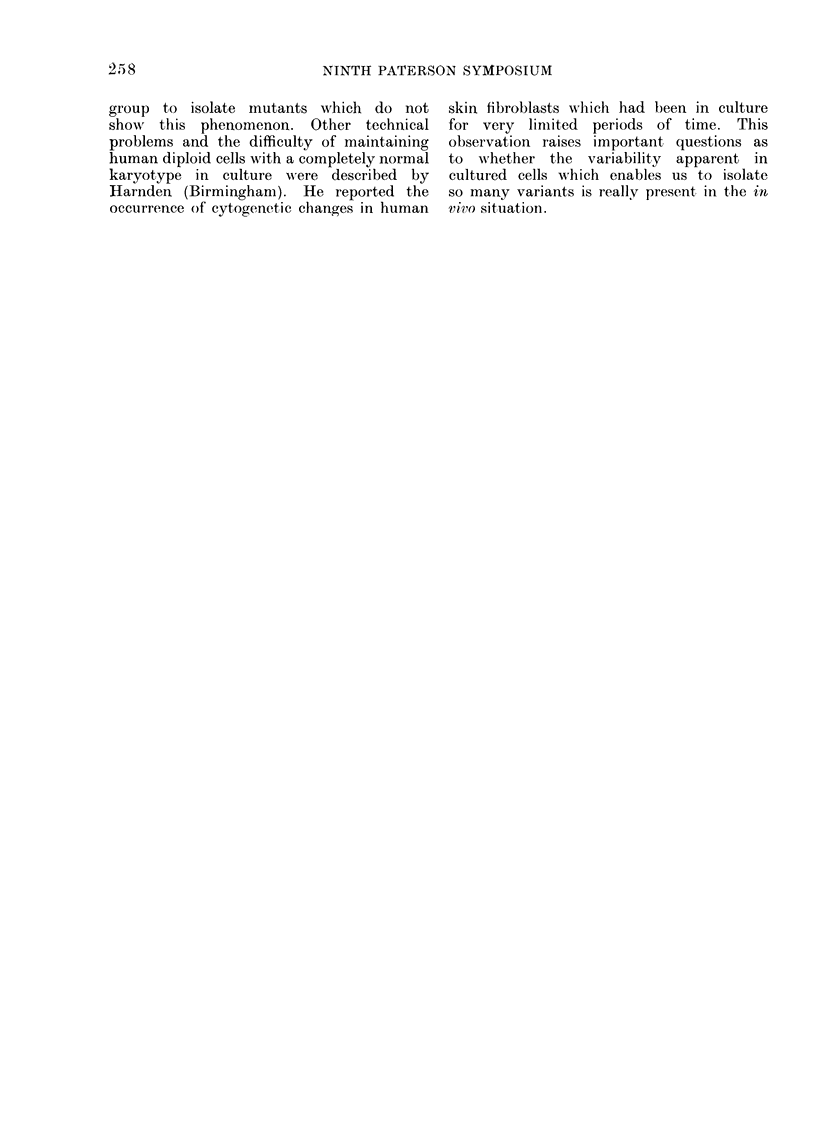# Ninth Paterson Symposium on Mechanisms of Mutagenesis in Somatic Mammalian Cells

**Published:** 1975-02

**Authors:** 


Br. J. Cancer (1975) 31, 255

### NINTH PATERSON SYMPOSIUM

### ON MECHANISMS OF MUTAGENESIS IN SOMATIC MAMMALIAN CELLS

The Ninth Paterson symposium was held at the **Paterson Laboratories, Christie Hospital and Holt Radium Institute, Manchester, from 17–19 October 1974** to discuss the current status of somatic cell genetics. There were 32 participants from seven countries. The symposium essentially dealt with mammalian cell lines and markers suitable for the study of mutation induction and the genetics and biochemistry of these and many other gene loci.

Cox (Harwell) described his technique using x-irradiated feeder layers which enable high plating efficiencies to be achieved with freshly isolated diploid embryo fibroblasts, and also his studies on x-ray induced mutations in such cells. Two genetic markers were used. Diploid human fibroblasts are normally unable to utilize fructose, but mutants with this ability arise spontaneously at a frequency of $1 \times 10^{-6}$. X-irradiation induced an increase in mutation frequency proportional to dose for this marker and for cells able to grow in the presence of high concentrations of 6-thioguanine. Dose response curves for mutation induction by x-rays at both these markers were cumulative, *i.e.* higher induction rates were observed at higher doses.

The current status of our knowledge of the genetics of Xeroderma Pigmentosum was reviewed by Bootsma (Rotterdam). Five complementation groups have now been defined and the biochemical characteristics of some of the groups are now known. Cell lines from three of the groups have been shown to be defective in ability to perform the incision step required for repair replication of u.v. irradiated DNA whilst a cell line from a fourth group is possibly exonuclease defective.

Further studies on human cells were reported by Maher (Detroit), who used resistance to 8-azaguanine as a marker to study relative sensitivity to mutation induction in normal human fibroblasts and in those derived from a variety of patients suffering from Xeroderma Pigmentosum (XP) which is known to result from a deficiency in excision repair capacity. Using ultraviolet light, 7-bromomethyl benz (a) anthracene and N-acetoxy-2 acetylamino flourene she demonstrated that in all cases cell lines from xeroderma patients showed greater sensitivity to cell killing, but when sensitivity to mutation induction was considered relative to survival, no enhancement of mutability resulting directly from the defect in excision repair was demonstrable, suggesting that excision repair is not error prone.

The data presented by a number of other speakers emphasized the fact that induced mutation frequencies appear to be more closely related to the way in which cells handle DNA damage than to the nature of the initial damage itself.

Dose response studies for a variety of chemical and physical mutagens in two cell lines, one mouse and one Chinese hamster, using two different genetic markers, resistance to excess thymidine and resistance to 8-azaguanine, were reported by Fox (Manchester). There was considerable uniformity in the response of these two cell-lines in terms of induced mutation frequencies for the different mutagens when frequencies were related to cell survival. In view of the known differences in amounts of excision repair induced by the mutagens used, the inference was, as in the work of Maher that excision repair in mammalian cells is probably an accurate process. Since induced mutation frequencies and cell lethality are closely related, and chromosome aberrations and cell lethality are also closely related, it seems likely that mechanisms by which chromosome aberrations and mutations arise are similar. This possibility was also discussed by Roberts (London) who described the enhancement of chromosome damage, mutation frequency and cell lethality by caffeine, which is known to inhibit post-replication repair processes. The possible involvement of such post-replication repair processes in the production of mutations was discussed extensively by this speaker who presented a number of models demonstrating the possible correlations between initial DNA damage by methylating agents, subsequent DNA replication on a damaged template, mutation and chromosome aberrations.

The elucidation of the interrelationships between post-replication repair processes, cell survival and mutation, should be greatly assisted by the study of an XP variant described by Bridges (Brighton) and Maher (Detroit). This variant which shows near normal sensitivity to u.v., and normal excision repair, is apparently defective in post-replication repair, and is more mutable than one would predict by comparing u.v. induced mutation frequencies at equitoxic doses. Bridges also discussed the inhibition of post-replication repair processes in bacterial systems by chloramphenicol. The component sensitive to inhibition is error prone, and the other, resistant to chloramphenicol is error free. The extension of such studies to mammalian cells should further clarify the role of repair processes in mutation induction.

The use of the V79 Chinese hamster cells and mutation to 8-azguanine resistance in the analysis of the mutagenic effectiveness of ionizing radiation of different LET was discussed by Munson (Harwell). The LET dependence of mutation induction in V79 cells was found to be similar to that for a forward mutation in *E. coli* which is thought to result from the production of double strand breaks.

An illustration of the use of V79 cells and 8-azaguanine resistance as a marker in the study of mutagenic effects of hydrocarbon carcinogens requiring metabolic activation was given by Huberman (Israel), who used irradiated feeder layers to metabolize such compounds. A correlation between numbers of cells in the feeder layer, *i.e.* amount of active metabolites produced and induced mutation frequency was observed. Here again, there was a close correlation between cytotoxicity and mutagenicity.

Data also indicating a close relationship between cell lethality and mutation induction was presented by Howard (Manchester) who described x-ray induced mutation in a completely different eucaryotic system, *i.e.* the spores of the fern *Osmunda regalis.* Here again modification of cell survival, *e.g.* by irradiation in oxygen, or dose fractionation, modified mutation frequency in parallel. Thus in a number of cell systems there is a close relationship between mutation frequency and cell killing for a number of mutagens.

The apparent similarity of response of the HGPRT locus in V79 cells and of the Tk locus in mouse lymphoma cells to chemical and physical mutagens may not apply when ouabain is used as a selective agent. Mutation to ouabain resistance in V79 cells and mouse lymphoma cells occurs spontaneously at a very low rate, undetectable in most cases, and is apparently dominant or co-dominant. Arlett (Brighton) reported significant induction of ouabain resistant mutants in V79 cells using a variety of chemical mutagens and u.v. With this marker, there seems to be greater differences between the mutant frequencies induced by various compounds which may be related to the dominance or co-dominance of mutations at this locus, or to the biochemical nature of the mutation itself.

A further exception was presented by Fahmy and Fahmy (London). Their studies of the mutational response of *Drosophilla melanogaster* to a wide variety of chemical carcinogens indicated no correlation between mutation and lethality, but did indicate a mutational specificity of potent carcinogens at the " bobbed " locus which specifies r DNA genes. A different approach to the detection of induced mutations in cultured human cell lines was described by Watson (London). Lymphoid cell lines were screened for 24–40 markers using gel electrophoresis techniques both under control situations and after exposure to the carcinogen n-methyl-n-nitrosoguanidine. This technique allows the screening of large numbers of loci and can detect deletions by loss of bands on electrophoretic gels, and also probably point mutations resulting in altered protein mobility. The great advantage of this technique is that protein modifications can be detected without the use of selective agents, which can under certain circumstances affect the response of the cell population as discussed by Simons (Leiden). Here, using human diploid cells, the type of mutant recovered depends strongly on the dose of selective agent (8-azaguanine) used.

For some time there has been much discussion as to the genetic nature of the drug resistant variants. Considerable progress in the characterization of variants resistant to 8-azaguanine, 8-azg$^r$, has been made by Caskey (Houston). Changes in the level of activity of the enzyme hypoxanthine guanine phosphoribosyl transferase (HGPRT) are known to be associated with 8-azaguanine and 6-thioguanine resistance. Two classes of mutants were described, those which possess positive HGPRT activity, and those which do not. By the use of a precipitation inhibition assay, mutants which do or do not possess altered enzyme proteins can be identified. Much of the data presented by this author strongly suggested that a considerable proportion of the 8-azg$^r$ mutants isolated represent gene mutations. This conclusion was confirmed by studies of spontaneous and induced reversion rates in a number of clones. In general frequencies of reversion were low $1 \times 10^{-7}$ to $1 \times 10^{-5}$. A warning was given about reports indicating that some 8-azg$^r$ clones have high reversion frequencies. In some cases these may not indicate true reversion rates since the HAT medium used for back selection may not completely prevent the growth of all 8-azg$^r$ clones. This has not been adequately controlled in some published reports. Further characterization of mutants at the HGPRT locus was described by Shin (New York) who also reported two technical developments which should facilitate progress in this field. The first, a rapid method for the identification of HGPRT allo-enzymes, involves electrophoresis on a cellulose acetate gel followed by detection of enzyme activity through a fluorescent end-product formed in a specific coupled reaction with inosine-5 monophosphate dehydrogenase. The second, involved the use of " nude " athymic mice. Cells of many origins will grow readily in such mice and thus the large numbers of cells required for the biochemical analysis of mutant enzymes can be easily and rapidly obtained. Evidence was also presented by this speaker which suggests that a particular chicken/mouse cell line in which all chick chromosomes have apparently been lost is capable of modulating its HGPRT activity depending whether it is grown in HAT medium or 8-azaguanine containing medium.

Chu (Michigan) presented a sophisticated study of mutant V79 cells which are gal$^-$, *i.e.* unable to utilize galactose. Genetic characterization of these mutants showed that the gal$^-$ mutation behaved as a recessive character and complemented the human chromosome $A_2$. Evidence was presented that alteration of structural genes was not involved, nor were the mutants defective in galactose transport. It was proposed that such mutants may be the result of a mutation at a regulator gene which interacts with more than one receptor site and positively controls enzyme synthesis.

Harris (Berkeley) described examples of phenotypic changes which occur in hybrids produced by conventional techniques, *i.e.* Tk$^-$ $\times$ HGPRT$^-$, but which cannot be related to chromosome loss as is usually the case in cell hybrids. Early segregants from these hybrids possessed the ability to grow in BUdR medium as well as in HAT medium. However, on subsequent subculture over a 300 day period the ratio of colonies capable of growth in HAT to those capable of growth in BUdR fell, indicating a loss of thymidine kinase enzyme which was also correlated with a changed ability to utilize thymidine. Such data which cannot be explained on the basis of gene mutation or chromosome segregation are suggestive of a slow shift in gene expression during clonal evolution possibly by epigenetic mechanisms. Further uses of cell hybrids in genetic analysis were described by Bodmer (Oxford) who was concerned mainly with the genetic analysis of the HL-A system. The serological determinants are membrane glyco-proteins consisting of 2 subunits, one of which is beta-2-microglobulin. The gene for beta-2 microglobulin has been assigned using somatic cell hybrids to chromosome 6, in contrast to the genes for the HL-A system which are on chromosome 15. A method whereby antibodies specific to one human chromosome, can be obtained was also described.

Goldfarb (Glasgow) described the selection and characterization of mutants affecting amino-acid transport and utilization which should be of great value in elucidating the genetics of growth control mechanisms and protein synthesis in mammalian cells.

A discussion of the technical problems involved in the isolation of various drug resistant mutants was provoked by the data presented by Slack (Glasgow) who clearly described the phenomenon of metabolic co-operation and the attempts of this group to isolate mutants which do not show this phenomenon. Other technical problems and the difficulty of maintaining human diploid cells with a completely normal karyotype in culture were described by Harnden (Birmingham). He reported the occurrence of cytogenetic changes in human skin fibroblasts which had been in culture for very limited periods of time. This observation raises important questions as to whether the variability apparent in cultured cells which enables us to isolate so many variants is really present in the *in vivo* situation.